# Biomarker-guided selection of intravesical therapy in high-risk non–muscle invasive bladder cancer: A contemporary review

**DOI:** 10.1177/17562872261485770

**Published:** 2026-09-02

**Authors:** Willian Ito, Isamu Tachibana

**Affiliations:** 1 Department of Urology, University of Texas Southwestern, Dallas, TX, USA

**Keywords:** non–muscle invasive bladder cancer, BCG-unresponsive disease, biomarkers, artificial intelligence, liquid biopsy

## Abstract

High-risk non–muscle invasive bladder cancer poses therapeutic challenges, with significant rates of recurrence and progression with standard intravesical bacillus Calmette-Guérin (BCG) therapy. Current surveillance strategies lack accurate risk stratification models to predict individual treatment response and personalized treatment options. Simultaneously, there are no well-validated alternatives to replace the current gold-standard approach based on clinical and pathologic features. This review examines emerging biomarkers and advanced technologies with the potential to enhance patient selection and personalize intravesical therapy in HR-NMIBC. Artificial intelligence(AI)-driven histopathologic tools, such as the computer histological AI biomarker, have demonstrated the ability to identify non-responders to standard therapy using whole-slide digital pathology images. In parallel, radiomics-enhanced imaging has shown promise in assessing tumor biology and immune microenvironment features predictive of BCG responsiveness. Liquid biopsy, especially urine tumor DNA analysis, is now available in the arsenal to detect minimal residual disease, stratify recurrence risk, and predict treatment response even before clinical or radiographic evidence of recurrence. Tissue-based genomic profiling has also revealed molecular alterations associated with treatment resistance, though additional validation is needed. Together, these next-generation biomarkers may represent a pivotal shift toward precision oncology in bladder cancer and their incorporation into NMIBC future clinical guidelines is both anticipated and necessary.

## Plain language summary

High-risk non–muscle invasive bladder cancer often comes back even after the gold-standard therapy, the so called intravesical BCG. Today, doctors can only learn whether BCG will work after a patient has already received it, which means some patients lose valuable time on a treatment that is unlikely to help them. Researchers are developing new biomarkers, which are tests that can give early clues about how tumors might behave. These tests may help doctors choose the right treatment sooner and avoid delays in care. This article reviews several promising types of biomarkers. One is an artificial-intelligence tool that studies tumor tissue under the microscope. It can spot patterns that suggest whether BCG is likely to work. Another approach uses advanced imaging—like special MRI or CT scans—to measure features inside and around the tumor that humans cannot see. These features may reveal how aggressive a tumor is or how it might respond to BCG. Scientists are also studying liquid biopsy, which looks for tumor DNA in urine. Because bladder tumors shed DNA directly into urine, this test may show early signs of cancer returning—even before symptoms or imaging changes appear. Tissue-based genetic testing is another area of progress, helping identify gene changes that may predict resistance to treatment. Together, these new tools could help doctors match each patient with the therapy that is most likely to work for them. Although more research is still needed, the growing evidence suggests that biomarker-guided care may soon become an important part of improving outcomes for people with high-risk bladder cancer.

## Introduction

Non–muscle invasive bladder cancer (NMIBC) represents about 70% of bladder cancer cases.^1^ High-risk NMIBC (HR-NMIBC) poses clinical challenges, with recurrence rates approaching 50% and progression in about 20% of patients within five years.^1,2^ Clinicians currently base their recommendations for intravesical therapy primarily on clinical risk. Bacillus Calmette-Guérin (BCG) remains the gold-standard treatment for high-risk disease.^3–5^ Still, up to 40% of patients may fail BCG. Additionally, 8-10% of individuals are simply unable to tolerate BCG, and ongoing shortages highlight the need for more precise treatment selection.^6–10^

BCG-unresponsive NMIBC represents a critical subgroup and current guidelines recommend early radical cystectomy (RC) or enrollment in clinical trials for bladder-sparing alternatives.^3–5,11^ Its natural history is normally aggressive and progression to muscle-invasive bladder cancer (MIBC) occurs in about 7% of these patients within 1 year and up to 25–39% by 5 years.^12^

An obvious but major clinical challenge is that BCG-unresponsive status is presently identified only *after* treatment failure, resulting in patients with very high-risk disease receiving therapy that is ineffective. This points to a critical unanswered question: can we identify these non-responders *before* BCG initiation, allowing for more effective upfront treatment?

Emerging biomarkers may offer a path toward more personalized care. Artificial intelligence (AI), advanced imaging techniques, and genomic biomarkers are actively being explored to predict treatment response and guide therapy selection.

This review summarizes the literature on these modalities and their potential role in refining intravesical therapy for NMIBC beyond traditional tools currently used for risk stratification.

## Methodology

A targeted literature review informed by the Preferred Reporting Items for Systematic Reviews and Meta-Analyses (PRISMA) principles was conducted to identify biomarkers predictive of response to intravesical therapy in patients with NMIBC. The search strategy utilized terms related to NMIBC, intravesical therapy, BCG, treatment response, and molecular, urinary, and tissue biomarkers. These terms were selected to focus the review on biomarkers capable of identifying patients with NMIBC at risk of non-response to intravesical therapy. Studies were considered eligible if they evaluated biomarker performance or reported predictive or prognostic outcomes related to intravesical BCG or alternative therapies in NMIBC.

Eligible study designs included original research articles, prospective and retrospective cohort studies, clinical trials, systematic reviews, and meta-analyses. Studies were excluded if they focused exclusively on muscle-invasive or metastatic bladder cancer without a specific NMIBC subgroup analysis, were non-English publications, conference abstracts without full-text availability, or lacked sufficient methodological detail.

Article titles and abstracts were independently screened by two authors, followed by full-text review of potentially eligible studies. Relevant data, including study design, patient population, biomarker type, sample size, primary outcomes, and reported limitations, were extracted and synthesized narratively.

## Review

### Can we predict BCG failure?

Historically, response to BCG has been attributed to both patient and tumor-related factors.^12^ Patient-related variables include age, gender, occupation, and tobacco use.^12,13^ Tumor-related characteristics, such as size, multifocality, presence of CIS, lymphovascular invasion (LVI), and histological variants can contribute to treatment outcomes.^14–16^ Specifically, presence of LVI alone, aggressive variants, or CIS combined with T1—including extensive T1 disease and persistence of T1 on re-TURBT—are generally associated with diminished chances to respond to additional intravesical therapy.^17–19^

Given these known associations, traditional risk stratification models rely on clinical and pathological features.^5,20,21^ Yet, these models are often criticized as they can overestimate the risk of recurrence and progression, leading to potential overtreatment.^22^ Previous attempts to utilize fluorescence *in situ* hybridization to predict BCG response have also failed, as this method suffers from poor sensitivity and nonquantitative readout.^23,24^ As a result, currently there are no features that can correctly predict which patients will experience long-term response following intravesical BCG.^25^

### AI-based biomarkers

A recent multicenter trial by Lotan et al. developed and validated an AI-based histopathology assay termed “*Computer Histological AI*” (CHAI) (Valar Labs; Palo Alto, CA), designed to predict clinical outcomes following BCG therapy in patients with HR-NMIBC. Using digital whole-slide images from TURBT specimens, CHAI analyzes subtle morphologic features, covering over 62 million µm^2^ of tissue to identify patterns associated with central clinical outcomes, such as recurrence, progression, BCG-unresponsiveness, and need for radical cystectomy.^26^

The model was developed on a cohort of 303 patients and subsequently validated in 641 individuals, demonstrating robust risk stratification. High-risk patients identified by CHAI had significantly lower RFS (HR 2.08, p < .0001), worse progression-free survival (PFS) (HR 3.87, p < .001), and higher cystectomy rates (HR 3.35, p < .001), independent of traditional clinicopathologic factors and established risk models such as EORTC 2016 and EAU 2021.^20,22^

CHAI illustrates the potential of computational pathology to improve risk stratification by providing a reproducible and timely method to identify patients unlikely to benefit from BCG. Within an acceptable turnaround time, this AI biomarker could generate a personalized risk profile indicating the likelihood of BCG failure, enabling both patients and clinical teams to tailor treatment accordingly.

Furthermore, Packiam et al. assessed how the same CHAI biomarker could help guide treatment beyond BCG. In a cohort of patients with HR-NMIBC treated with either BCG or sequential intravesical gemcitabine and docetaxel (gem/doce), patients with CHAI high-risk signature were significantly associated with poor outcomes in the BCG group but not in those receiving intravesical chemotherapy. Notably, the RFS at 24 months was superior in patients marked as CHAI high-risk and treated with intravesical gem/doce compared to BCG (84% versus 70%, respectively; p < 0.02).^27^

Although still in early stages, these results may have important clinical implications. By identifying patients less likely to benefit from BCG, the CHAI biomarker supports avoidance of BCG or prompt treatment escalation for patients marked with high-risk signature.

### Artificial intelligence, deep-learning, and radiomics in NMIBC

Similarly with the development of AI-based biomarkers, there is a growing interest in the integration of AI, deep learning (DL), and radiomics for advanced oncologic diagnosis.^28,29^ Radiomics is a quantitative imaging technique that extracts high-dimensional features (e.g., intensity, shape, texture, and spatial patterns) from a large image database to characterize tumor phenotypes and develop predictive and prognostic models.^30^

Initial indications have suggested that combining radiomics with multiparametric MRI (mpMRI) may offer valuable insights into tumor grade and overall recurrence risk in NMIBC. Li et al. developed a radiomics-clinical nomogram using mpMRI-derived features and clinical variables (age, tumor count) to predict tumor grade prior to initial TURBT, achieving a high validation AUC of 0.931, which outperformed clinical data alone.^31^ Similarly, Huang et al. showed that a preoperative mpMRI-based DL clinical model significantly improved prediction of RFS (C-index 0.832). This model utilized radiomic features assessing intratumoral and peritumoral areas, which were independently associated with recurrence risk.^32^ These findings suggest that mpMRI-based radiomics may capture tumor heterogeneity and biological behavior not so evident in standard clinical assessment, which may help to identify higher-risk patients preoperatively. As such, mpMRI-radiomics models could support more tailored decision-making in NMIBC, enabling clinicians to better select subsequent interventions for patients at higher risk of progression in a timely fashion.

### Radiomics to predict BCG response

Following this rationale, the proposed ability of radiomics to stratify the risk of BCG recurrence essentially lies in detecting features imperceptible to human capacities.^30^ In a recent study, Ye et al. applied non-negative matrix factorization (NMF), a method previously used in oncology to decompose complex data into component parts that capture distinct biological patterns.^33^ Particular to bladder cancer, the authors used NMF to break down radiomic features from small lesions detected on contrast-enhanced CT images obtained prior to index TURBT. This model—also trained on a development cohort and tested an external validation cohort—accurately predicted 9 of 12 BCG failures and 60 of 92 non-failures, with an AUC of 0.79 in the development set and 0.70 in the validation set for predicting BCG failure at one year. Limitations include its retrospective design, the relatively small number of BCG failures in the development cohort, and the need to manually capture the region of interest on each individual scan (i.e., it is not an automated process).^34^ While not without shortcomings, this approach highlights the potential of radiomics as a predictive tool for BCG outcomes, with the prospect of guiding treatment selection as methodologies evolve in the future.

From a different perspective, Krpina et al. explored how radiomics could indirectly reveal the microscopic characteristics of the tumor microenvironment (TME) and its potential influence on BCG response.^35^ It is known that the immunological TME in bladder cancer is shaped by various immune cells, among which tumor-infiltrating lymphocytes (TILs) play a pivotal role in regulating the immune response to cancer.^35^ The therapeutic effect of BCG likely stems from its ability to induce a local immune cascade, leading to the accumulation of immune cells that secrete cytokines and chemokines, which in turn promotes the recruitment of TILs into the tumor stroma.^36^ Consequently, the presence of TILs after BCG therapy could be seen as a marker of effective immune activation. In this context, a novel radiomics-based biomarker has been proposed to predict TIL-status and response to BCG therapy using features also extracted from contrast-enhanced CT scans obtained prior to TURBT along with correspondent clinicopathological data. In their multicenter cohort of 206 patients, the radiomics TIL-based biomarker (Rad_TIL_) model showed a good predictive value, with an AUC of 0.846 for identifying TIL status and AUC of 0.728 for predicting BCG response. Notably, the majority patients with high Rad_TIL_ scores were more likely to respond to BCG (approximately 60.9% responded to BCG), highlighting the potential of this new biomarker to refine treatment planning.^37^

As contrast-enhanced CT scan is routinely used in the standard evaluation of bladder cancer based on most clinical guidelines, integrating radiomics represents a logical and promising advancement in the ongoing evolution of bladder cancer management.

### Tissue-based genomic testing

Following TURBT, the availability of tumor tissue may offer a crucial opportunity to obtain detailed genomic information to then identify useful biomarkers with predictive potential. Francesca et al. evaluated a multigene next-generation sequencing (NGS) panel in a retrospective cohort of 19 patients with HR-NMIBC and found that BCG-responsive tumors (10 patients) frequently harbored mutations in receptor tyrosine kinases (*FGFR3, ALK, DDR2*), *CTNNB1* and *PIK3CA*.^38^ Interestingly, this contrasts with previous findings that linked *FGFR3* mutations to a “cold” TME—characterized by diminished immune cells in the tumor stroma—and BCG resistance, suggesting a more complex relationship between mutation status and treatment response.^39^ On the non-responder side, over 50% of tumors lacked detectable mutations within the panel, though 33% (3/9 patients) did have *TP53* mutations,^38^ which have been previously associated with a poorer prognosis.^40^ However, *TP53* mutations were also found in 30% of the responders.^38^ While these results indicate that NGS profiling could help with patient-physician therapeutic decisions, the small sample size limits generalizability, and the presence of overlapping mutations across response groups highlights the need to assess specific mutation combinations rather than overall mutation burden.

Therefore, more recent research has increasingly focused on identifying unambiguous genomic and immune markers with predictive potential. *HER2* overexpression, for example, has been linked to poor outcomes and is frequently observed in high-grade tumors, MIBC, and metastases.^41^ In a retrospective study of 454 patients treated with BCG, high immunohistochemical *HER2* expression was associated with significantly lower 3-year RFS (39.3% vs. 74.2%) and higher risk of progression or metastasis (37.6% vs. 3.4%). Their group also reported an alarming 83.5% 5-year relapse rate when high *HER2* was found in HR-NMIBC. Similarly, genomic alterations such as *ARID1A* mutations and *CCNE1* amplification have been linked to increased relapse risk following BCG treatment. While driver mutations like *TERT* are often conserved, other clinically relevant genes—including *FGFR3*, *PIK3CA*, *TSC1*, and *TP53*—frequently shift over time, suggesting that relapse may be driven by the evolution of persistent tumor subclones.^42^ In parallel, immune-related biomarkers are gaining traction. Turdo et al. identified *IDO1*, an immunosuppressive enzyme upregulated by IFN-γ produced by TILs, as a potential predictor of BCG failure. Using RNA sequencing in T1 patients, they found high *IDO1* expression was associated with poorer BCG outcomes.^43^

Collectively, these findings highlight the promise of genomic profiling in tailoring intravesical therapy. While early results are encouraging, prospective validation, technical standardization, and cost-effectiveness analysis will be essential before widespread clinical adoption.

### Liquid biopsy

Liquid biopsy has quickly become a transformative tool in precision oncology, offering a non-invasive approach to detect and monitor tumor-specific genetic changes through circulating biomarkers. A key focus is the analysis of cell tumor DNA (ctDNA), which is a subset of cell-free DNA released into body fluids during tumor cell apoptosis.^43,44^

Plasma-based liquid biopsy, particularly through the analysis of ctDNA in the serum, has already shown utility in the context of MIBC and the detection of systemic disease. Serum ctDNA may be effective in identifying MRD and predicting recurrence following definitive local therapy in MIBC, with the latent potential to guide adjuvant systemic treatment.^45,46^

In NMIBC, urine-based liquid biopsy—particularly the analysis of urine tumor DNA (utDNA), a form of ctDNA—may offer a more sensitive method for detecting tumor-derived signals. The direct contact between the bladder and urinary tract enables tumor DNA to be shed directly into urine, making it an accessible and effective medium for monitoring persistent or recurrent disease. Given the presumably limited systemic ctDNA dissemination in NMIBC, utDNA assays may provide improved sensitivity for surveillance and monitoring of local treatment response.

### Urine-based liquid biopsy

An important study by Scott et al. provided foundational evidence supporting the clinical utility of utDNA in the management of HR-NMIBC treated with BCG. This investigation demonstrated that NGS could be successfully applied to urine supernatants and sediments to detect somatic mutations associated with urothelial cancer. Remarkably, the study found a significantly higher cumulative mutation rate in urine-derived DNA compared to plasma, highlighting the biological rationale for prioritizing urine as the primary liquid biopsy medium in localized bladder cancer.^47^

Subsequent findings have reinforced the clinical relevance of utDNA in identifying patients at risk for poor outcomes in the setting of BCG-unresponsive NMIBC. A notable study by Rac et al. evaluated urinary comprehensive genomic profiling (uCGP) using the UroAmp™ assay (Convergent Genomics) to assess recurrence risk following intravesical BCG. Urine was collected before and after TURBT and post-BCG, with utDNA mutations tracked against tumor tissue. Among 33 patients, 13 (39%) experienced recurrence, with significantly higher genomic disease burden (GDB) observed in these individuals. uCGP stratification revealed a 24-month RFS of 45% in high-risk patients versus 100% in low-risk (p = 0.009). While 60% of high-risk patients recurred over the full surveillance period (28 months), only one low-risk patient had recurrence demonstrated. Notably, those in the top GDB quartile (i.e., 75 to 100% percentile) had RFS of 0%.^48^

Furthermore, in a prospective biomarker analysis embedded within the SWOG S1605 trial, which studied the role of atezolizumab in BCG-unresponsible HR-NMIBC, researchers also utilized the UroAmp™ assay to investigate the potential role of utDNA during therapy. Baseline utDNA positivity was strongly associated with inferior clinical outcomes—patients with detectable utDNA had a substantially lower complete response rate (13% vs. 71%) and reduced 18-month event-free survival (23% vs. 51%) compared to those who were utDNA negative. Importantly, even among patients who were clinically disease-free at 3 months, persistent utDNA positivity identified a subgroup with significantly higher risk of progression.^49^

In this way, patients with positive utDNA levels during or shortly after BCG therapy could be identified before clinical evidence of disease. This early detection may support timely consideration of alternative interventions or enrollment in clinical trials investigating novel therapeutic approaches. Conversely, patients with negative utDNA might avoid unnecessary treatment intensification. As technology and validation studies progress, the integration of utDNA monitoring into standard surveillance protocols could also fundamentally transform the management of HR-NMIBC.

## Comparative summary of emerging biomarkers in HR-NMIBC

Table 1. Provides a comparative overview of the key studies discussed in this review, including study design, sample size, primary outcomes, and major limitations. This synthesis highlights important differences in evidence quality across biomarker modalities and underscores areas requiring prospective validation.

**Table 1.** Comparative summary of key biomarker studies in HR-NMIBC.

Biomarker category	Study (author, year)	Design	N	Key outcomes	Major limitations
AI Digital Pathology	Lotan et al., 2025	Retro. multicenter (dev/val)	944	CHAI high-risk: worse RFS (HR 2.08), PFS (HR 3.87), higher cystectomy rate (HR 3.35); independent of EORTC/EAU risk models	Retrospective; no prospective validation; commercial assay (cost/access); limited generalizability to non-digital pathology settings
AI Digital Pathology	Packiam et al., 2025	Retro. cohort	NR	CHAI high-risk: RFS 84% vs. 70% at 24 months favoring gem/doce over BCG (p<0.02); predictive of differential treatment benefit	Retrospective; non-randomized treatment groups; selection bias; requires prospective validation in head-to-head trial
Radiomics (mpMRI)	Huang et al., 2025	Retro. cohort	NR	DL-radiomics model: C-index 0.832 for RFS prediction; intratumoral and peritumoral features independently predictive	Retrospective; no external validation; MRI not universally available; requires standardized acquisition protocols
Radiomics (CT-BCG prediction)	Ye et al., 2022	Retro. dev/ext. val.	NR	AUC 0.79 (dev)/0.70 (val) for predicting BCG failure at 1 year using CT radiomics; correctly predicted 9/12 BCG failures	Small BCG-failure cohort; manual ROI segmentation (non-automated); retrospective; limited generalizability
Radiomics (TIL-based)	Chen et al., 2025	Retro. multicenter	206	RadTIL: AUC 0.846 for TIL status; AUC 0.728 for BCG response; ∼60.9% high-RadTIL patients responded to BCG	Retrospective; CT protocol variability; surrogate TIL endpoint; no direct BCG response randomization
Tissue Genomics (NGS)	Francesca et al., 2025	Retro. cohort	19	BCG responders: FGFR3, ALK, CTNNB1, PIK3CA mutations; TP53 in 33% of non-responders but also 30% of responders	Very small sample (n=19); overlapping mutation profiles; no external validation; limited generalizability
Tissue Genomics (IHC/HER2)	Bacon et al., 2022	Retro. cohort	454	High HER2: 3-yr RFS 39.3% vs. 74.2%; 83.5% 5-yr relapse in HR-NMIBC; ARID1A and CCNE1 also associated with relapse	Retrospective; IHC standardization concerns; no prospective BCG-stratified analysis; lacks therapeutic implication data
Liquid Biopsy (utDNA)	Rac et al., 2024	Prospective biomarker study	33	uCGP high-risk: 24-mo RFS 45% vs. 100% low-risk (p=0.009); top GDB quartile: RFS 0%; uCGP stratifies recurrence risk	Small sample; single assay platform; no treatment-modification arm; requires prospective validation with larger cohorts
Liquid Biopsy (utDNA)	St-Laurent et al., 2025 (SWOG S1605)	Prospective (embedded biomarker)	NR	utDNA+ at baseline: CR 13% vs. 71%; 18-mo EFS 23% vs. 51%; persistent utDNA+ predicts progression even in clinical CR	Single-arm trial; atezolizumab-specific context; single assay platform; needs validation across other therapies

AI = artificial intelligence; AUC = area under the curve; BCG = bacillus Calmette-Guérin; CHAI = Computer Histological AI; CR = complete response; CT = computed tomography; dev = development cohort; DL = deep learning; EFS = event-free survival; ext. val. = external validation; GDB = genomic disease burden; HR = hazard ratio; IHC = immunohistochemistry; mpMRI = multiparametric MRI; NGS = next-generation sequencing; NR = not reported; PFS = progression-free survival; RadTIL = radiomics tumor-infiltrating lymphocyte model; RFS = recurrence-free survival; retro. = retrospective; ROI = region of interest; TIL = tumor-infiltrating lymphocyte; uCGP = urinary comprehensive genomic profiling; utDNA = urine tumor DNA; val. = validation cohort.

**Figure 1.**
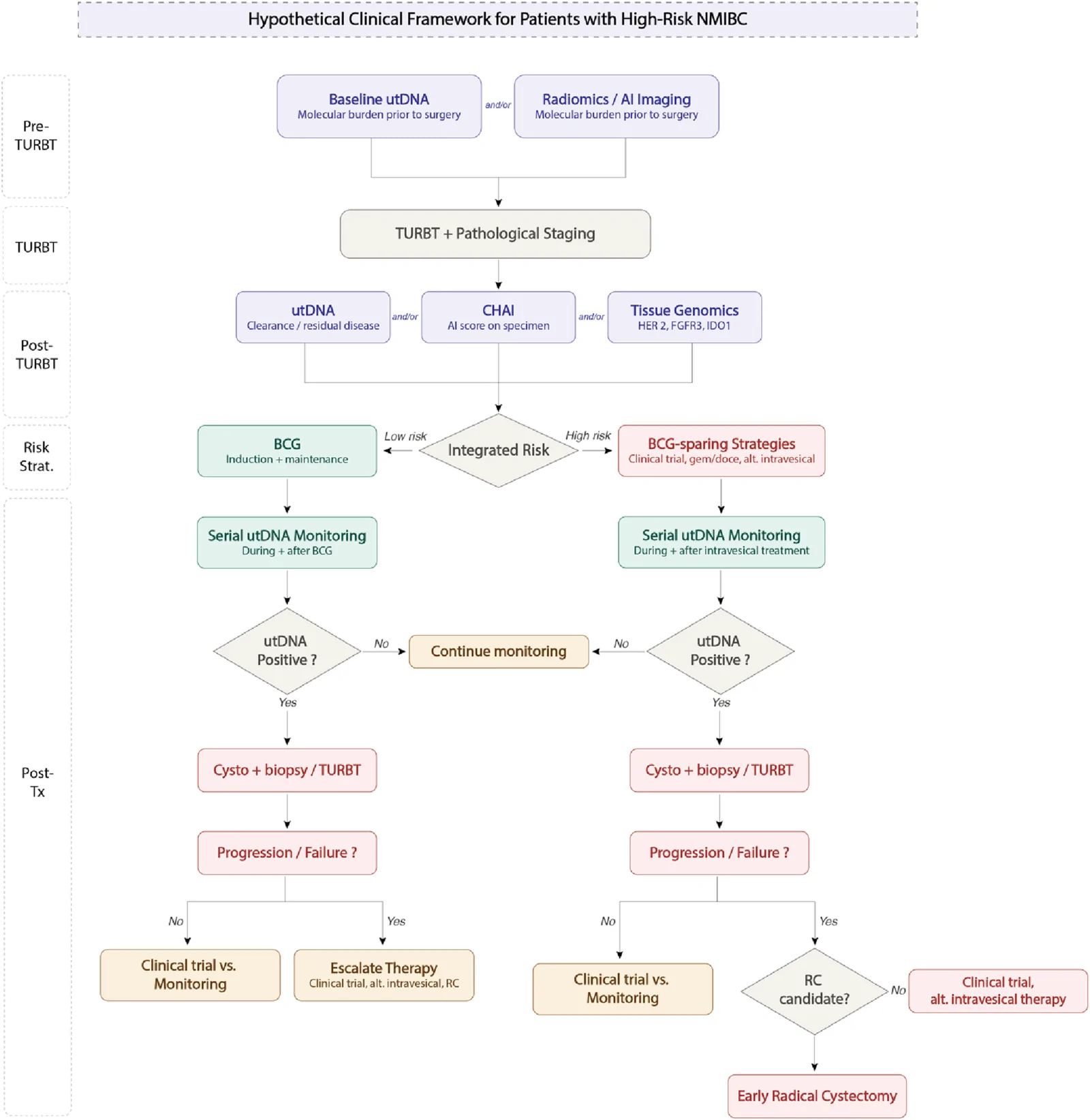
Hypothetical clinical framework for patients with high-risk NMIBC.

A hypothetical biomarker-guided treatment algorithm for newly diagnosed HR-NMIBC integrates non-invasive and tissue-based biomarkers across the disease course – Figure 1.

Prior to TURBT, patients may undergo non-invasive biomarker assessment with utDNA analysis. mpMRI with radiomics/DL-based assessment may be considered selectively when there is concern for high-grade T1 disease with possible occult muscle invasion or other features suggestive of T2 disease. Following TURBT, residual disease risk may be further assessed using specimen-dependent modalities, including post-operative utDNA, the combined histologic and CHAI score, and/or tissue genomic profiling, depending on availability and clinical context. These data are then integrated to generate a biomarker-based risk classification. Patients classified as biomarker low-risk receive standard BCG therapy, whereas biomarker high-risk patients are counseled regarding a lower likelihood of BCG response and may be considered for BCG-sparing approaches such as alternative intravesical therapies or clinical trials. Serial utDNA monitoring is then performed in all patients. Persistent negative utDNA supports continued surveillance, while positive utDNA prompts diagnostic evaluation with cystoscopy and/or repeat TURBT. Confirmed progression results in treatment escalation, including alternative intravesical therapy, clinical trial enrollment, or RC, with earlier consideration of cystectomy in biomarker high-risk patients. This framework remains purely hypothetical and requires prospective validation before clinical implementation.

### Future directions

By synthesizing inputs such as CHAI histology scores, utDNA profiles, and radiomic risk models, such tools may transform HR-NMIBC care into a dynamic, microenvironment-informed precision strategy. Further refinement of each of these modalities holds promise to improve real-time, individualized treatment options.

The development of novel therapies for NMIBC is increasingly guided by risk stratification, with several ongoing trials focused on the BCG-naïve setting. For example, the PIVOT-006 trial is evaluating adjuvant intravesical cretostimogene grenadenorepvec vs. surveillance in intermediate-risk BCG-naïve patients. In the high-risk setting, the BRIDGE trial is prospectively comparing standard BCG to intravesical gem/doce. Notably, the recent phase 3 CREST trial demonstrated improved outcomes with the addition of the PD-1 inhibitor sasanlimab to BCG in high-risk BCG-naïve patients. However, the added benefit of combination therapy underscores the need to better identify patients most likely to respond—highlighting the potential role of predictive biomarkers. Combining data generated from these trials with novel biomarkers may help identify optimal candidates for non-BCG therapies, ultimately supporting more precise, risk-adapted treatment strategies in NMIBC.

## Conclusion

Biomarker-guided strategies will likely redefine the management of HR-NMIBC, particularly in optimizing intravesical therapy selection. Although still evolving, tools such as the CHAI biomarker, radiomics-enhanced imaging, and liquid biopsy technologies are converging to enable more precise risk stratification and individualized treatment planning. By identifying patients unlikely to respond to BCG prematurely, these innovations offer an opportunity to pivot to alternative therapies or timely cystectomy, potentially improving outcomes and reducing delays in care.

Initial evidence suggests these biomarkers are not merely complementary to standard approaches but may fundamentally change clinical decision-making. The integration of computational histologic signatures, molecular burden indices, and radiomic models with traditional clinical and pathological features may be accelerated with further advances in AI. While larger prospective trials are needed to validate and standardize these technologies, the growing body of evidence unveils their role as next-generation biomarkers in HR-NMIBC. Their integration into practical clinical guidelines is not a distant goal, but an anticipated and necessary evolution in care.

## Data Availability

As a review article, all data and materials are available.*

## Appendix

### List of abbreviations

AI: Artificial intelligence
ALK: Anaplastic lymphoma kinase
AUC: Area under the curve
BCG: Bacillus Calmette-Guérin
CHAI: Computer Histological AI
CIS: Carcinoma in situ
CR: Complete response
CT: Computed tomography
ctDNA: Circulating tumor DNA
DL: Deep learning
EAU: European Association of Urology
EFS: Event-free survival
EORTC: European Organisation for Research and Treatment of Cancer
GDB: Genomic disease burden
HR-NMIBC: High-risk non–muscle invasive bladder cancer
IDO1: Indoleamine 2,3-dioxygenase 1; IFN-γ: Interferon-gamma
IHC: Immunohistochemistry
LVI: Lymphovascular invasion
MIBC: Muscle-invasive bladder cancer
MRI: Magnetic resonance imaging
mpMRI: Multiparametric MRI
MRD: Minimal residual disease
NGS: Next-generation sequencing
NMF: Non-negative matrix factorization
NMIBC: Non–muscle invasive bladder cancer
PD-1: Programmed cell death protein 1
PFS: Progression-free survival
RadTIL: Radiomics tumor-infiltrating lymphocyte model
RC: Radical cystectomy
RFS: Recurrence-free survival
SUO: Society of Urologic Oncology
TIL: Tumor-infiltrating lymphocyte
TME: Tumor microenvironment
TURBT: Transurethral resection of bladder tumor
uCGP: Urinary comprehensive genomic profiling
utDNA: Urine tumor DN.
